# Efficacy of immune‐checkpoint inhibitors in metastatic gastric or gastroesophageal junction adenocarcinoma by patient subgroups: A systematic review and meta‐analysis

**DOI:** 10.1002/cam4.3417

**Published:** 2020-09-01

**Authors:** Yulia Kundel, Michal Sternschuss, Assaf Moore, Gali Perl, Baruch Brenner, Hadar Goldvaser

**Affiliations:** ^1^ Davidoff Cancer Center Beilinson Hospital Rabin Medical Center Petah Tikva Israel; ^2^ Sackler Faculty of Medicine Tel Aviv University Tel Aviv Israel

**Keywords:** gastric cancer, gastroesophageal cancer, immune‐checkpoint inhibitors, immunotherapy

## Abstract

**Background:**

Efficacy of immune checkpoint inhibitors (ICIs) in metastatic gastric/gastroesophageal junction (GEJ) adenocarcinoma is inconsistent. Whether the efficacy of ICIs is comparable across different subgroups remains unknown.

**Methods:**

We identified randomized controlled trials (RCTs) that compared standard treatment for metastatic gastric/GEJ adenocarcinoma to ICIs. Hazard ratios (HRs) and 95% confidence intervals (CI) for overall survival (OS) were extracted and pooled in a meta‐analysis. Prespecified subgroups were included as follows: age at randomization (</≤65 vs ≥/>65 years), gender (female vs male), ethnicity (Asians vs non‐Asians), performance‐status (0 vs 1), tumor location (gastric vs GEJ), and histological subtype (diffuse vs others). OS in patients with programmed death ligand (PD‐L1) positive and with microsatellite instability‐high (MSI‐H) were also extracted and pooled in a meta‐analysis.

**Results:**

Five RCTs comprising 2,264 patients were analyzed. Compared to standard therapy, ICIs did not improve OS (HR = 0.86, 95% CI 0.71‐1.03, *P* = .10) and the effect of ICIs on OS was similar in all subgroups. Nonsignificantly greater effect sizes were seen in older patients (HR = 0.85 vs 0.88, *P = *.81), male (HR = 0.82 vs 0.99, *P* = .16), Asians (HR = 0.86 vs 0.96, *P* = .55), performance‐status 0 (HR = 0.84 vs 0.88, *P* = .81), GEJ tumors (HR = 0.78 vs 0.90, *P* = .37), and nondiffuse subtype (HR = 0.71 vs 0.79, *P* = .62). ICIs were associated with significantly improved OS in patients with MSI‐H (HR = 0.33, *P* = .001), but not in PD‐L1 positive disease (HR = 0.86, *P* = .06).

**Conclusions:**

Compared to standard treatment, ICIs in metastatic gastric/GEJ adenocarcinoma did not improve OS. None of the evaluated subgroups has shown increased magnitude of effect to ICIs, aside of the small group with MSI‐H tumors.

## Graphical abstract

Compared to standard treatment, ICIs in metastatic gastric/GEJ adenocarcinoma did not improve OS. None of the evaluated subgroups including, age, gender, ethnicity, performance status, primary tumor location, and histological subtype, has shown increased magnitude of effect from ICIs. In exploratory analysis for small group with MSI‐H tumors, treatment with ICIs was associated with significant OS improvement compared to the control group.

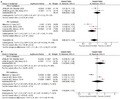

## 1 INTRODUCTION

Most gastric and gastroesophageal junction (GEJ) cancers present at an advanced stage and have poor prognosis with a median survival of approximately 1 year.^1^ Cytotoxic chemotherapy is the backbone of treatment in these cancers.^2^, ^3^ First‐line chemotherapy most commonly comprises a platinum and a fluoropyrimidine combination.^3^, ^4^ Other agents such as taxanes or irinotecan have shown only modest activity, usually with a short duration of response after progression on first line chemotherapy.^5^, ^6^ During the last decade several nonchemotherapy treatments for gastric cancer have emerged, such as trastuzumab which has shown improved overall survival (OS) in patients with human epidermal growth factor receptor 2 (HER2) positive disease when added to standard chemotherapy.^7^ However, HER2‐positive disease represents only 15% of whole gastric cancer patients.^7^

In recent years, immune‐checkpoint inhibitors (ICIs) have redefined the treatment paradigm of various types of advanced cancers.^8^, ^9^, ^10^, ^11^ In gastric and GEJ adenocarcinoma, early phase studies have shown that treatment with ICIs has an overall manageable toxicity profile and encouraging efficacy results.^12^, ^13^, ^14^, ^15^ Randomized controlled trials (RCTs) investigating the role ICIs in gastric and GEJ adenocarcinoma demonstrated inconsistent results,^16^, ^17^, ^18^, ^19^, ^20^, ^21^, ^22^ and biomarkers to predict response to ICIs in this population have not been well‐defined. Pembrolizumab, an anti programed cell death protein 1 (PD‐1) inhibitor, is currently approved by the US Food and Drug Administration (FDA) for patients with metastatic gastric or GEJ adenocarcinoma whose tumors express programmed death‐ligand 1 (PD‐L1), defined as Combined Positive Score [CPS] ≥1, with disease progression on or after two or more prior lines of therapy.^23^ This approval was supported by the results of the KEYNOTE‐059 trial that demonstrated relatively high response rate with durable response to pembrolizumab monotherapy in these patients.^14^ The detection rate of PD‐L1 expression in gastric cancer tissues is highly variable, ranging between 14%–69%.^24^ PD‐L1 expression is associated with worse outcomes and adverse histo‐pathological features, including more advanced T stage, nodal involvement, venous invasion, Epstein‐Barr virus infection, and microsatellite instability‐high (MSI‐H) status.^24^, ^25^ However, while PD‐L1 expression might be an important biomarker to predict response to ICIs in other solid tumors,^11^, ^26^, ^27^ in gastric and GEJ adenocarcinoma, PD‐L1 expression alone may not be an adequate biomarker.

In solid tumors with MSI‐H, single‐agent pembrolizumab has shown robust activity, including in heavily pretreated patients,^28^, ^29^ leading to an FDA approval of pembrolizumab monotherapy in tumors with MSI‐H disease, regardless of tumor's primary site.^23^ In metastatic gastric and GEJ adenocarcinoma specifically, MSI‐H is associated with encouraging activity of ICIs. Unfortunately, only a small minority of gastric or GEJ adenocarcinoma patients have evidence of MSI‐H.^16^, ^17^

Geographical regions have a well‐established impact on gastric and GEJ adenocarcinoma outcome, with improved OS in Asians compared to non‐Asians. The favorable outcome in Asians persists after adjusting for disease stage.^30^ Ethnicity has also been shown to affect response to different systemic treatments,^31^, ^32^, ^33^ with data suggesting distinct molecular entities by geographical region.^30^ However, the impact of ethnicity on response to ICIs remains unclear.

Here, we report on a meta‐analysis evaluating the magnitude of effect of ICIs compared to standard treatment on the outcomes of patients with metastatic gastric or GEJ adenocarcinoma. We also aimed to identify whether specific subgroups, defined by clinical and pathological features, have differential effect from ICIs.

## 2 MATERIALS AND METHODS

### 2.1 Literature Review and Study Identification

A literature search utilizing MEDLINE (Host: PubMed) and EMBASE identified RCTs published between January 2010 and December 31 2019 which explored the benefit of ICIs (either as monotherapy or as a combination of with chemotherapy) compared to standard treatment in upper gastrointestinal malignancies including gastric, GEJ and esophageal adenocarcinoma. The following search algorithm was used: (((gastric cancer[MeSH Terms]) OR gastroesophageal cancer[MeSH Terms]) OR esophageal cancer[MeSH Terms]) AND ((immunotherapy OR PD‐1 inhibitor OR PDL‐1 inhibitor OR anti PD‐1 OR anti PDL‐1 OR nivolumab OR opdivo OR pembrolizumab OR keytruda OR atezolizumab OR tecentriq OR avelumab OR bavencio OR durvalumab OR imfinzi OR immune checkpoint)). The EMBASE search was in title/abstract without Mesh Terms. To improve the sensitivity, we also searched databases from the Annual Meetings of the American Society of Clinical Oncology (ASCO), the European Society of Medical Oncology (ESMO), ASCO gastrointestinal and ESMO gastrointestinal meetings during the last 4 years (2017 to 2020) and reviewed citation lists. Eligibility criteria were as follows: studies which included patients with metastatic gastric and/ or GEJ adenocarcinoma and investigated ICIs with either PD1 or PDL1 inhibitors. Comparisons between ICIs monotherapy or combination of chemotherapy and ICIs and chemotherapy to standard of care (either chemotherapy or best supportive care if prior two lines of chemotherapy were given) were allowed. Treatment could be first line of subsequent line for advanced setting. Studies were included only if outcomes on progression‐free survival (PFS), OS or both were reported. The search was restricted to English language only. Both randomized phase 2 and phase 3 trials were allowed.

### 2.2 Data extraction

Hazard ratios (HRs) and 95% confidence intervals (CIs) of the effect of the treatment with ICIs compared to standard treatment on PFS, OS and OS by subgroups were extracted. Data on PFS by subgroups were not reported. Prespecified subgroups included patients’ age at time of randomization (categorized as age </≤ 65 years vs ≥/>65 years), gender (female vs male), ethnicity (Asians vs non‐Asians), Eastern Cooperative Oncology Group performance status (ECOG PS) (0 vs 1), primary tumor location (gastric vs GEJ), and histological subtype (diffuse vs other subtypes). Additionally, data on OS in patients with MSI‐H and PD‐L1‐positive disease were collected. We also extracted data on number of included patients, median duration of follow‐up, regimens given in the experimental and the control arms and whether treatment was first line or more advanced. When available, data on the proportion of patients with HER2 positive and MSI‐H were also extracted.

Data were extracted independently by two reviewers (MS and HG). Discrepancies were resolved by consensus. All data were extracted from primary publications, their associated online appendices or from the conference presentation if the study was yet to be published.

### 2.3 Data synthesis and statistical analysis

The extracted HRs and CIs from individual studies were pooled in a meta‐analysis using generic inverse variance and random effects modeling. To evaluate the effect of ICIs by subgroups, HRs for each subgroup were presented for descriptive purposes and differences between the subgroups were assessed using methods describes by Deeks *et al* ^34^ Analyses were performed using RevMan 5.3 (The Cochrane Collaboration, Copenhagen, Denmark). Statistical heterogeneity was reported using Cochran Q and I^2^ statistics. Statistically significant heterogeneity was defined as a Cochran Q *P* < .10 or I^2^ greater than 50%. Due to substantial clinical heterogeneity between studies, analyses were performed using random effects modeling irrespective of statistical heterogeneity. Analysis was performed if data were available for at least three studies.

Multiple sensitivity analyses were performed on OS and PFS to evaluate the effect of ICIs as first line treatment (rather than other settings), the effect of PD‐L1 inhibitor (rather than PD‐1 inhibitor), placebo in the control group (rather than chemotherapy) and excluding a study which included Asians only. These prespecified categories were also used to explored the potential sources of heterogeneity of included studies. The interaction between PD‐1 inhibitors and PD‐L1 inhibitors in the intention to treat population was also assessed using methods describes by Deeks *et al* ^34^ Statistical significance was defined as *P* < .05.

## 3 RESULTS

**Figure 1:**
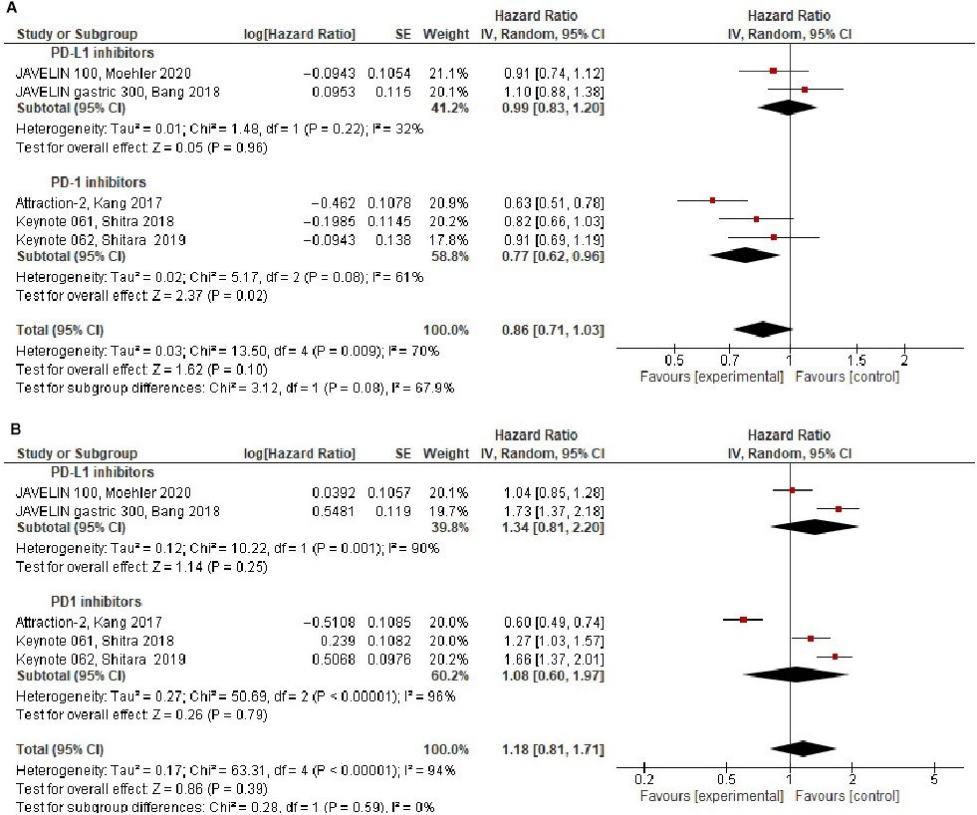
Study selection

The search identified 1771 records. After exclusions, seven publications reporting outcomes on five trials were included,^16^, ^17^, ^18^, ^19^, ^20^, ^21^, ^22^ see Figure 1. For one study, a post hoc analysis for Asians and patients with CPS ≥ 10 was presented at the recent 2020 ASCO annual meeting,^35^ but the relevant extracted results were identical the these previously published.^17^ Overall, eligible studies comprised 2,264 patients. Individual study characteristics and quality assessment of included studies are shown in Table 1 and Table 2, respectively. In one trial, data were extracted only on patients with CPS ≥ 1 (excluding patients with CPS < 1) as the primary outcome was defined for these patients.^16^ In another trial, there were two investigational arms: pembrolizumab monotherapy and a combination of pembrolizumab with chemotherapy. However, data on subgroups were only available for the comparison between pembrolizumab monotherapy and chemotherapy.^17^ One study included Asians only ^18^, ^19^ and this was the only study that used placebo in the control group rather than chemotherapy. Otherwise, patients’ characteristics with regard to gender, age, ECOG PS, primary tumor location and histological subtype were similar between included studies. One study evaluated ICIs as first line,^17^ other study evaluated maintenance avelumab compared to chemotherapy in patients who did not progress after 1st line of chemotherapy ^22^ and the other trials used ICIs in more advanced disease.^16^, ^18^, ^20^ Two studies used anti‐PD‐L1 ^20^, ^22^ and three studies used anti‐PD‐1.^16^, ^17^, ^18^

**Table 1:** Characteristics of included studies

Trial/ Median follow‐up	Study arm (num patients)	Control arm (num patients)	Line of treatment	Median Age (years)	Asians (%)	Male (%)	ECOG PS = 0 (%)	Primary location: stomach (%)	Diffuse subtype (%)	Other
ATTRACTION‐2, Kang 2017,^18^, ^19^ (8.8 months)	Nivolumab 3 mg/kg q 2‐week (330)	Placebo (163)	≥3	62	100%	71%	29%	83%	34%	PDL1 positive: 14%
JAVELIN gastric 300, Bang 2018 ^20^ (10.6 months)	Avelumab 10mg/kg q 2‐week (185)	paclitaxel 80 mg/m^2^ d1,8,15 OR irinotecan 150 mg/m^2^ q 4‐week OR BSC^c^ (186)	3	60	25%	72%	35%	70%	21%	PDL1 ≥ 1%: 27%
KEYNOTE 061, Shitra 2018 ^16^, ^a^ (7.9 months)	Pembrolizumab 200 mg q 3‐week (196)	paclitaxel 80 mg/m^2^ d1,8,15 week (199)	2	62.5	30%	74%	46%	66%	23%	CPS ≥ 1%: 100% HER2 +: 19% MSI‐H^*^: 5%
KEYNOTE 062, Shitara 2019,^17^, ^21^, ^b^ (11.3 months)	Pembrolizumab 200 mg q 3‐week (256)	Placebo + cisplatin 80 mg/m^2^ + 5FU 800mg/m2 d1‐5 OR capecitabine twice a day 1‐14 q 3‐week (250)	1	62	24%	71%	49%	71%	45%	CPS ≥ 1%: 100% HER2 +: 0% MSI‐H: 7%
JAVELIN gastric 100, Moehler 2020^22^	Avelumab 10 mg/kg q 2‐week (249)	Oxaliplatin + 5FU+LCV/ capecitabine OR BSC^d^ (250)	Maintenance after 1st line without progression	61.5	23%	66%	42%	71%	‐	PDL1 ≥ 1%: 12% CPS ≥ 1%: 22%^d^, ^e^ HER2 +: 0% MSI‐H: 3%

Abbreviations: 5FU, 5‐fluorouracil; BSC, best supportive care; ECOG PS, Eastern Cooperative Oncology Group performance status; HER2, human epidermal growth factor receptor 2; LCV, leucovorin; MSI‐H, microsatellite instability—high; PDL1, programmed death ligand.

^a^ Only the patients with CPS ≥ 1 were included.

^b^ Data from the experimental arm with pembrolizumab monotherapy were included.

^c^ Three patients received BCS only.

^d^ Choice of chemotherapy of BCS decided by investigators prior randomization, a total pf 19 patients did not receive chemotherapy.

^e^ Stratification to PDL1 + was done using the 73‐10 pharmDx assay (Dako), postexploratory analysis assessment of CPS was done with 22C3 pharmDx assay (Dako).

**Table 2:** Quality assessment of included studies

	Random Sequence Generation	Allocation Concealment	Blinding of participants and personnel	Blinding of outcome assessment	Incomplete outcome data	Selective reporting
ATTRACTION‐2, Kang 2017^18^, ^19^	Low	Unclear	Low	Low	Low	Low
JAVELIN gastric 300, Bang 2018^20^	Low	Low	Low	Low	Unclear	Low
KEYNOTE 061, Shitra 2018^16^	Low	Low	Unclear	Low	Unclear	Low
KEYNOTE 062, Shitara 2019 ^17^, ^21^, ^a^	Unclear	Unclear	Unclear	Low	Low	Unclear
JAVELIN gastric 100, Moehler 2020 ^22^, ^a^	Unclear	Unclear	Low	Low	Unclear	Unclear

^a^ As the KEYNOTE 062 have and the JAVELIN gastric studies have been presented only in a form of abstracts/ oral presentations and the full manuscripts have been published yet, data for quality assessment are limited.

### 3.1 Intention to treat

**Figure 2:**
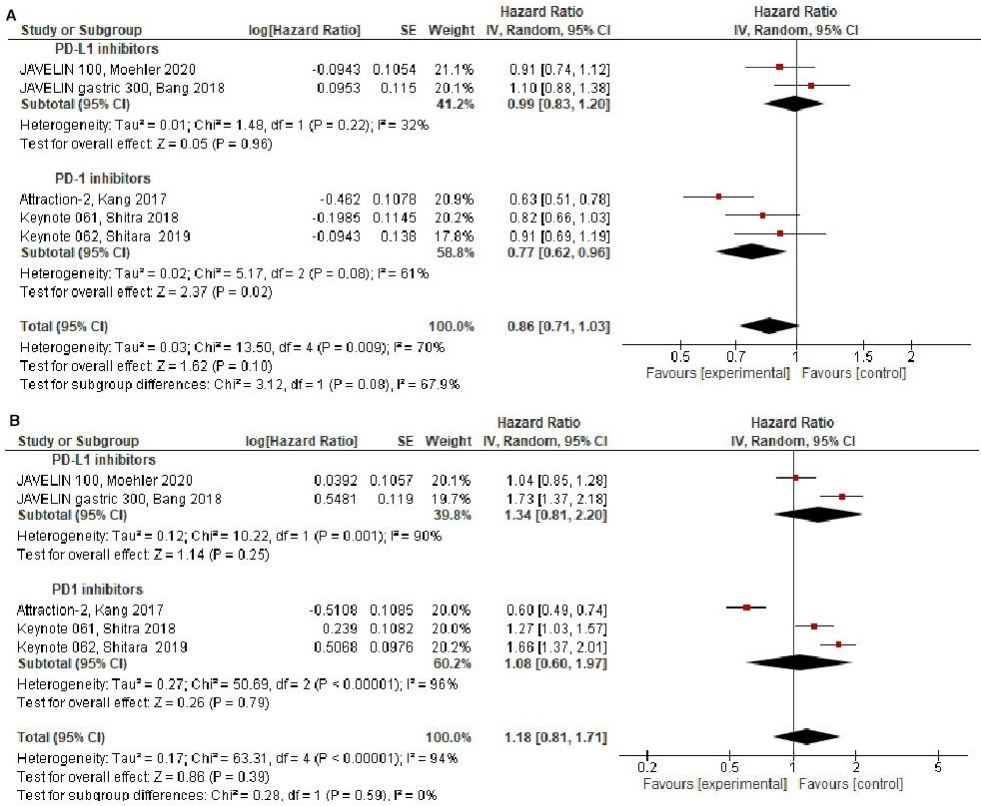
Forest plots in the intention to treat population for: (A) Overall‐survival, (B) Progression‐free survival

Overall, compared to standard therapy, treatment with ICIs did not improve OS (HR = 0.86, 95% CI 0.71‐1.03, *P* = .10), see Figure 2A. There was statistically significant heterogeneity for OS (Cochran's *Q* *P = *.009, *I* ^2^ = 70%). In subgroup analysis by ICIs type, the magnitude of benefit for PD‐1 inhibitors was higher compared to PD‐L1 inhibitors (HR = 0.77, 95% CI 0.62‐0.96 vs. to HR = 0.99, 95% 0.83‐1.20) and approached statistically significance (p value for the subgroup difference = 0.08). Other sensitivity analyses for OS showed similar results (Table 3). Assessment of heterogeneity showed that excluding the ATTRACTION‐2 study, which compared ICIs to placebo and included Asians only, contributed to heterogeneity. Excluding this study resulted in nonsignificant heterogeneity and a similar effect size on OS (HR 0.93, 95% CI 0.82‐1.05, Cochran's *Q* *P = *.33, *I* ^2^ = 12%).

**Table 3:** Sensitivity analyses

Outcome	Primary analysis	Excluding study in 1st line treatment	Excluding studies with PDL1 inhibitor	Excluding study that included only Asians/ compared to placebo
OS (HR, 95% CI)	0.86 (0.71‐1.03)	0.85 (0.67‐1.06)	0.77 (0.62‐0.96)	0.93 (0.82‐1.05)
Heterogeneity for OS Cochran's Q (p* *value, *I* ^2^)	*P = *.009, *I* ^2^ = 70%	*P = *.004, *I* ^2^ = 77%	*P = *.08, *I* ^2^ = 61%	*P = *.33, *I* ^2^ = 12%
PFS (HR, 95% CI)	1.18 (0.81‐1.71)	1.08 (0.70‐1.66)	1.08 (0.60‐1.97)	1.39 (1.10‐1.76)
Heterogeneity for PFS Cochran's Q (*P *value, *I* ^2^)	*P < *.001, *I* ^2^ = 94%	*P < *.001, *I* ^2^ = 94%	*P < *.001, *I* ^2^ = 96%	*P = *.002, *I* ^2^ = 80%

Abbreviations: CI, confidence interval; HR, hazard ratio; OS, overall survival; PDL, programed death ligand; PFS, progression free survival.

PFS was comparable in ICIs group compared to standard therapy in the intention to treat population PFS (HR = 1.18, 95% CI 0.81‐1.71, *P* = .39), see Figure 2B. There was statistically significant heterogeneity for PFS (Cochran's *Q* *P < *.001, *I* ^2^ = 94%). Assessment of heterogeneity, did not identify a study that contributes to heterogeneity (Table 3). PFS was affected by excluding the ATTRACTION‐2 study (HR = 1.18 with all studies compared to HR = 1.39 after exclusion). Other sensitivity analyses for PFS showed similar results (Table 3).

With regard to toxicity, overall ICIs monotherapy had favorable toxicity profile compared to chemotherapy,^16^, ^17^, ^20^, ^21^, ^22^ but there were more adverse events for combination of chemotherapy and ICIs compared to chemotherapy.^17^, ^21^ When ICIs treatment was compared to placebo, there were more adverse events in the investigational arm.^18^

### 3.2 Ethnicity

**Figure 3:**
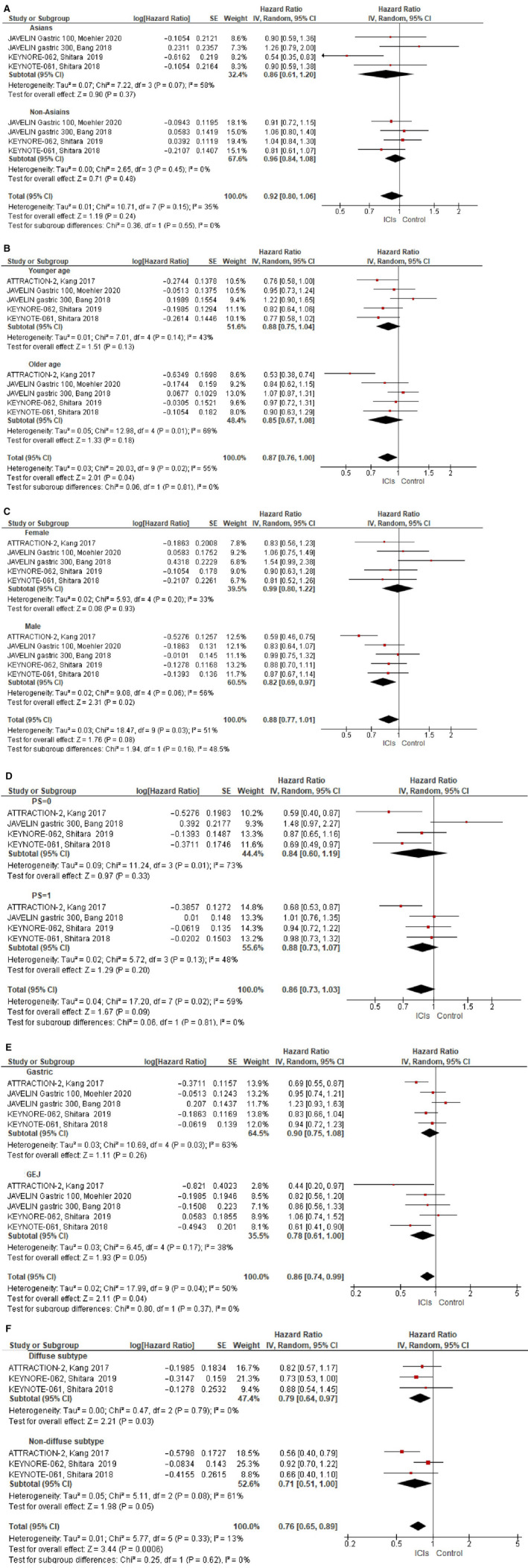
Forest plots for overall‐survival according to: (A) Ethnicity (Asians vs non‐Asians), (B) Age (≤/<65 vs>/≥65), (C) Gender (female vs male), (D) ECOG performants status (0 vs 1), (E) Primary tumor location (gastric vs GEJ), (F) Histological subtype (Diffuse subtype vs nondiffuse subtype). Hazard ratios for each trial are represented by the squares, the size of the square represents the weight of the trial in the meta‐analysis, and the horizontal line crossing the square represents the 95% confidence interval. The diamonds represent the estimated pooled effect. All *P* values are two‐sided

Four studied reported on OS by ethnicity.^16^, ^17^, ^20^, ^22^ The magnitude of benefit from ICIs on OS was higher in Asians (HR = 0.86, 95% CI 0.61‐1.20) compared to non‐Asians (HR = 0.96, 95% CI 0.84‐1.08), but this difference was not significant (subgroup difference *P* = .55), see Figure 3A. There was no statistically significant heterogeneity (Cochran's *Q* *P = *.15, *I* ^2^ = 35%), see supplementary Table 1. Pooled analysis of the data on Asians from these studies together with the ATTRACTION‐2, which included Asians only, did not show statistically significant impact of ICIs on OS in this population (HR = 0.79, 95% 0.61‐1.03, *P* = .09, Cochran's *Q* *P = *.03, *I* ^2^ = 61%).

### 3.3 Age

All studies reported on OS for younger age (<65 or ≤ 65) compared to older age (>65 or ≥ 65).^16^, ^17^, ^18^, ^20^, ^22^ OS was comparable in younger age (HR = 0.88, 95% CI 0.75‐1.04) compared to older age (HR = 0.85, 95% 0.67‐1.08), subgroup difference *P* = .81, see Figure 3B. There was statistically significant heterogeneity (Cochran's *Q* *P = *.02, *I* ^2^ = 55%), see supplementary Table 1.

### 3.4 Gender

All studies reported on OS by gender.^16^, ^17^, ^18^, ^20^, ^22^ The magnitude of benefit from ICIs was higher in men (HR = 0.82, 95% 0.69‐0.97) compared to women (HR = 0.99, 95% 0.80‐1.22), but this difference was not statistically significant (subgroup difference *P* = .16), see Figure 3C. There was statistically significant heterogeneity (Cochran's *Q* *P = *.01, *I* ^2^ = 51%). Assessment of heterogeneity showed that excluding the studies investigating PD‐L1 inhibitors (rather than PD‐1 inhibitor) and excluding the ATTRACTION‐2 study, contributed to heterogeneity, but this had no impact of the subgroup difference (p for the subgroup difference = 0.54 after exclusion of anti PD‐L1 inhibitors studies and *P* = .28 after exclusion of the ATTRACTION‐2 study), see Table S1.

### 3.5 Performance status

Four studies reported on OS by ECOG PS.^16^, ^17^, ^18^, ^20^ The effect of ICIs in patients with PS = 0 was comparable to the effect in patients with PS = 1 (HR = 0.84, 95% 0.60‐1.19 and HR = 0.88, 95% 0.73‐1.07, subgroup difference *P* = .81), see Figure 3D. There was statistically significant heterogeneity (Cochran's *Q* *P = *.02, *I* ^2^ = 59%). Assessment of heterogeneity showed that excluding the study investigating PD‐L1 inhibitors and excluding the ATTRACTION‐2 study, contributed to heterogeneity, but this had no impact of the subgroup difference (p for the subgroup difference = 0.35 after exclusion of PD‐L1 inhibitor study and *P* = .88 after exclusion of the ATTRACTION‐2 study), see Supplementary Table 1.

### 3.6 Primary tumor location

All studied reported on OS by primary tumor location.^16^, ^17^, ^18^, ^20^, ^22^ The magnitude of benefit was higher in patients with tumors in the GEJ (HR = 0.78, 95% CI 0.61‐1.00) compared to those with gastric tumors (HR = 0.90, 95% CI 0.75‐1.08), but this difference was not statistically significant, (subgroup difference *P* = 0. 37), see Figure 3E. There was statistically significant heterogeneity (Cochran's *Q* *P = *.04, *I* ^2^ = 50%). Assessment of heterogeneity showed that excluding the ATTRACTION‐2 study, contributed to heterogeneity, but this had no impact of the subgroup difference (p for the subgroup difference = 0.28), see Table S1.

### 3.7 Histological subtype

Three studies reported on OS by histological subtype.^16^, ^17^, ^18^ ICIs had a similar effect on diffuse histological subtype (HR = 0.79, 95% CI 0.64‐0.97) and nondiffuse subtypes (HR = 0.71, 95% 0.51‐1.00), subgroup difference *P* = .62, see Figure 3F. There was statistically significant heterogeneity (Cochran's *Q* *P = *.33, *I* ^2^ = 13%), see supplementary Table 1.

### 3.8 MSI‐H and PDL‐1 status

**Figure 4:**
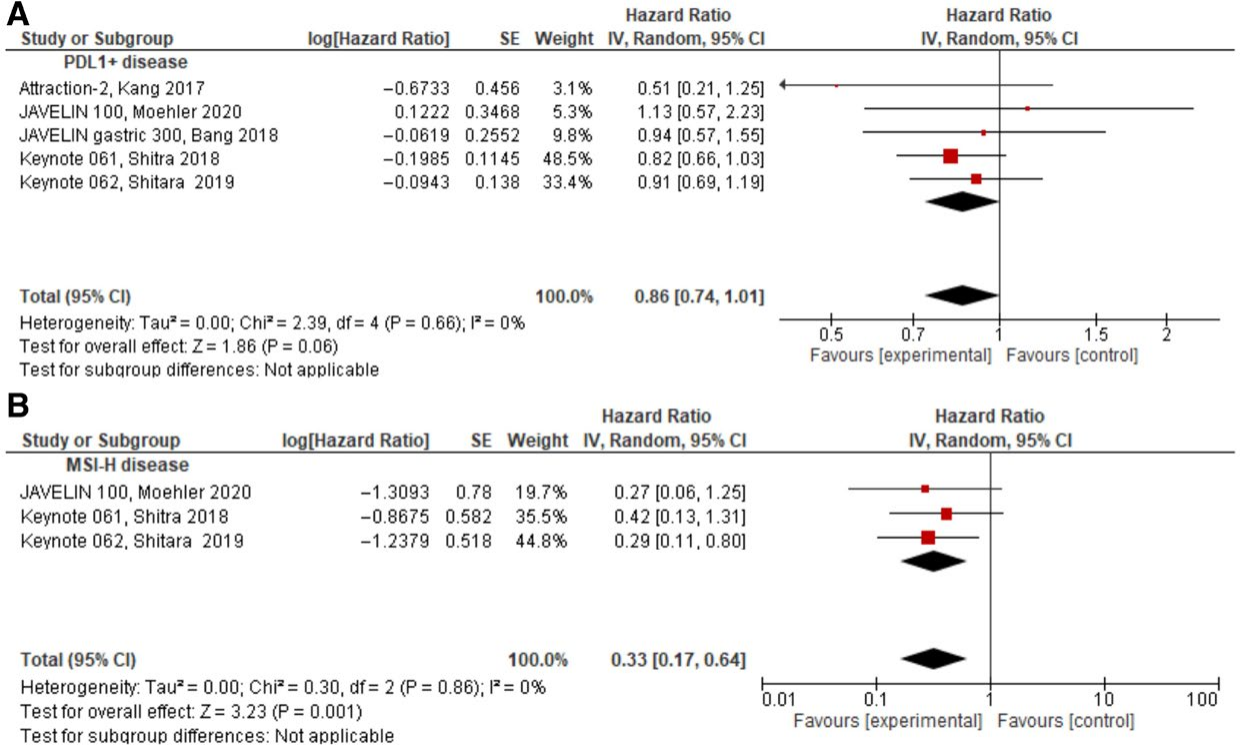
Forest plots for OS in: (A) MSI‐H disease, (B) PDL1‐positive disease

Three studies reported on OS for patients with MSI‐H.^15^, ^21^, ^22^ In these patients, ICIs were associated with a significant improvement is OS (HR = 0.33, 95% CI 0.17‐0.64, *P* = .001) (Figure 4A). On note, as OS results for mismatch repair‐proficient (MMRp) patients were not reported, the interaction between these subgroups could no be accounted for.

Data on OS for patients with PD‐L1‐positive disease were pooled from all studies.^16^, ^17^, ^18^, ^20^, ^22^ Compared to the control group, in patients with PD‐L1‐positive disease as prespecified by each study, treatment with ICIs approached a statistically significant improvement in OS (HR = 0.86, 95% CI 0.74‐1.01, *P* = .06), see Figure 4B. Of note, in one study, an exploratory analysis evaluated OS also by PD‐L1 status using a different assay than the assay used during enrolment (22C3 pharmDx rather than 73‐10 pharmDx assay [Dako]).^22^ When pooling the results from this exploratory analysis, treatment with ICIs was associated with a statistically significant improvement in OS (HR = 0.83, 95% CI 0.71‐0.96, *P* = .01). As data on PD‐L1‐negative tumors were insufficient and in two of the included studies all patients were PD‐L1‐positive,^16^, ^17^ the interaction between PDL1 positive to PDL1 negative could not be assessed.

## 4 DISCUSSION

Treating metastatic gastric and GEJ adenocarcinoma is often challenging due to low clinical benefit and short duration of response with standard treatment. The success of immunotherapy in other types of cancer ^8^, ^9^, ^10^, ^11^ and promising results for early phase studies in gastric cancer,^12^, ^13^, ^14^, ^15^ have generated tremendous interest in treating gastric and GEJ adenocarcinoma with ICIs, leading to several pivotal trials investigating the effect of ICIs in different settings, both in combination with chemotherapy or as monotherapy.^16^, ^17^, ^18^, ^19^, ^20^, ^21^, ^22^

In this meta‐analysis we aimed to investigate the efficacy of ICIs compared to standard treatment in metastatic gastric or GEJ cancer. We also aimed to identify whether a specific subgroup of patients would have a larger magnitude of benefit from ICIs. Overall, compared to standard treatment, ICIs did not improve PFS or OS. Importantly, the difference in OS between PD‐L1 inhibitors and PD‐1 inhibitors approached significance (HR = 0.99 and 0.77, respectively, p for the subgroup difference 0.08), suggesting possible improved efficacy of PD‐1 inhibitors in gastric or GEJ adenocarcinoma, though this difference was not statistically significant. Comparisons between the evaluated subgroups showed similar effect of ICIs and no specific subgroup was identified to benefit more from ICIs. In several of the evaluated subgroups there is higher magnitude of OS benefit in the ICIs arm (eg, for men HR = 0.82, 95% 0.69‐0.97, for diffuse subtype HR = 0.79, 95% CI 0.64‐0.97, and for GEJ tumors HR = 0.78, 95% CI 0.61‐1.00), however, as the difference between the reciprocal subgroups was not statically significant, these findings can not be account as statistically significant. Multiple sensitivity analyses did not identify a definitive subgroup that is expected to benefit more from ICIs.

Wang el al. previously published meta‐analysis on the efficacy and toxicity of ICIs in gastric or GEJ tumors, showing no OS benefit from ICIs in these patients.^36^ This meta‐analysis comprised five studies, but only three were RCTs and OS data were available only from two of these studies. In our meta‐analysis, efficacy results from 5 RCTs were pooled. Consistent with this previous meta‐analysis,^36^ no OS benefit from ICIs was seen. Additionally, here we presented detailed subgroup analyses by several important clinical and pathological characteristics.

In patients with MSI‐H disease, ICIs was associated with a significant improvement in OS. While patients with MSI‐H disease represent only a small minority of patients included in this analysis, and there were no data to compare the effect of treatment to patients with MMRp disease, the magnitude of effect for MSI‐H disease in this analysis was robust (HR = 0.33, *P* = .001) and consistent with prior reports of ICIs in tumors with MSI‐H,^28^, ^29^ bolstering the benefit from ICIs in this population.

PD‐L1‐positivity has an important role in predicting response to ICIs in some cancers ^11^, ^26^, ^27^ but not in others.^8^, ^37^ In gastric or GEJ cancer, initial approval for pembrolizumab was given for patients with PD‐L1‐positive disease, based on encouraging results from a single arm study.^14^ In our meta‐analysis, treatment with ICIs compared to standard treatment approached significance (HR = 0.86, 95% CI 0.74‐1.01, *P* = .06), but the magnitude of effect was similar to the effect in all patients (HR = 0.86, 95% CI 0.71‐1.03, *P* = .10). As data on efficacy for PD‐L1‐negative disease were insufficient, and two studies included only patients with PD‐L1‐positive disease, the interaction between OS in PD‐L1‐positive and ‐negative could not be assessed. Therefore, the conclusions that can be drawn for ICIs in PD‐L1‐positive patients from this meta‐analysis are limited. Additionally, PD‐L1 positivity was determined by different assays and methods in the included studies, a fact that further limits this analysis.

Gastric cancer in Asians represents a distinct clinical entity,^20^, ^21^, ^22^, ^23^, ^24^, ^25^, ^26^, ^27^, ^28^, ^29^, ^30^, ^31^, ^32^, ^33^ and therefore, ethnicity in gastric cancer is an important stratification factor and its influence on ICIs' efficacy should be determined. All patients in the ATTRACTION‐2 study, that was included in this meta‐analysis, were Asians and it was the only study that showed significant improvement in OS with nivolumab compared to the control group.^18^ However, in our meta‐analysis, the difference between Asians and non‐Asians was not significant (p for the subgroup *P* = .55). A hint for potential impact of ethnicity on response to ICIs may exists in the PFS results. A sensitivity analysis excluding the ATTRACTION‐2 study, showed a meaningful difference in PFS, demonstrating inferiority to ICIs when excluding this study (HR = 1.39 compared to 1.18). However, as this study was also the only one to use placebo in the control group (rather than chemotherapy), rapid progression and OS difference could be easily explained. Interestingly, in the PFS analysis there is significant statistical heterogeneity and this finding was noted also after excluding the ATTRACTION‐2. Other factors that might impact the heterogeneity might include different line of therapy or variability in efficacy of the investigated ICIs.

This study has several limitations. First, this is a literature‐based rather than an individual patient‐based meta‐analysis. Consequently, it is subject to publication bias. Second, the designs of the included studies in this meta‐analysis are heterogeneous with varied settings of treatment, types of ICIs and treatment in the control groups. Third, there is also heterogeneity in studies’ populations. Two studies only included patients with PD‐L1 positive tumors. Additionally, the methods to assess PD‐L1 positivity were inconsistent in the included studies. Of note, to address this heterogeneity, analyses were performed using random effects modeling (rather that fixed effect modeling) and multiple sensitivity analyses were performed. Finally, data on treatment post progression, including exposure to ICIs, are lacking. This might also affect OS.

In conclusion, in this meta‐analysis, compared to the control group, addition of ICIs to standard treatment did not improve outcome in gastric and GEJ cancer patients. With the exception of the small group of patients with MSI‐H disease, our analysis did not identify subgroups with greater statistically significant benefit on OS from ICIs. Further investigation of PD‐1 inhibitors rather than PD‐L1 inhibitors might be preferred, considering our findings in the subgroup analysis for OS by ICIs type. As treatment of metastatic gastric cancer has unmet needs given the poor OS with standard treatment, additional efforts should be made to better identify subpopulations that could benefit from ICIs.

## CONFLICT OF INTEREST

Dr Moore declared *honorarium* payment from MSD and Roche, all outside the submitted manuscript. Dr Brenner declared personal speaker and consulting fee from MSD, Roche, BMS, Merck Serono, AbbVie, Boehringer Ingelheim, traveling grants from MSD, Roche, Merck Serono, fees supporting clinical trials and scientific projects from Roche, BMS, Merck Serono, Sanofi, Oncotest‐Teva, all outside the submitted manuscript. Dr Goldvaser declared *honorarium* payment from Roche, Pfizer, Novartis, Oncotest, all outside the submitted manuscript. The other authors have no conflicts of interest to declare.

## AUTHOR CONTRIBUTIONS

Yulia Kundel: Data curation, investigation writing—original draft, writing—review and editing. Michal Sternschuss: Data curation, writing—review and editing. Assaf Moore: Validation, writing—review and editing. Gali Perl: Validation, writing—review and editing. Baruch Brenner: investigation, writing—review and editing. Hadar Goldvaser: conceptualization, Data curation; Formal analysis; methodology, investigation, project administration, supervision, writing—original draft,review, and editing.

## Supporting information

Table S1

## Data Availability

The data that support the findings of this study are available from the corresponding author upon reasonable request.
