# Supplementary material for: Efficacy of immune‐checkpoint inhibitors in metastatic gastric or gastroesophageal junction adenocarcinoma by patient subgroups: A systematic review and meta‐analysis
Source: Cancer Med. 2020 Sep 1;9(20):7613–25. doi: 10.1002/cam4.3417 (PMC7571828; doi:10.1002/cam4.3417)
Supplement: Supplementary file 1 — Table S1 [file CAM4-9-7613-s001.docx]

**Supplementary Table 1: Assessment of heterogeneity in the subgroups analyses Sensitivity analyses**

|  |  | **Primary analysis** | **Excluding study in 1^st^ line treatment** | **Excluding studies with PDL1 inhibitor** | **Excluding study that included only Asians/ compared to placebo** |
| --- | --- | --- | --- | --- | --- |
| Subgroup analysis by ethnicity | P value for the subgroups difference | 0.55 | 0.60 | 0.30 | - |
|  | Heterogeneity Cochran’s Q (p value, *I*^2^) | p = 0.15 *I*^2^ = 35% | p = 0.62, *I*^2^ = 0% | p = 0.13, *I*^2^ = 44% | - |
| Subgroup analysis by age | P value for the subgroups difference | 0.81 | 0.61 | 0.80 | 0.54 |
|  | Heterogeneity Cochran’s Q (p value, *I*^2^) | p = 0.02, *I*^2^ = 55% | p = 0.007, *I*^2^ = 64% | p = 0.03, *I*^2^ = 57% | p*=* 0.01, *I*^2^ = 62% |
| Subgroup analysis by gender | P value for the subgroups difference | 0.16 | 0.18 | 0.54 | 0.28 |
|  | Heterogeneity Cochran’s Q (p value, *I*^2^) | p = 0.03, *I*^2^ = 51% | p = 0.01, *I*^2^ =62% | p = 0.20, *I*^2^ =32% | p = 0.38, *I*^2^ =7% |
| Subgroup analysis by performance status | P value for the subgroups difference | 0.81 | 0.90 | 0.35 | 0.88 |
|  | Heterogeneity Cochran’s Q (p value, *I*^2^) | p = 0.02, *I*^2^ = 59% | p = 0.005, *I*^2^ = 70% | p = 0.14, *I*^2^ = 40% | p = 0.15, *I*^2^ = 38% |
| Subgroup analysis by primary tumor location | P value for the subgroups difference | 0.37 | 0.13 | 0.63 | 0.28 |
|  | Heterogeneity Cochran’s Q (p value, *I*^2^) | p = 0.04, *I*^2^ = 50% | p = 0.002, *I*^2^ = 58% | p = 0.09, *I*^2^ = 47% | p = 0.18, *I*^2^ = 31% |
| Subgroup analysis by histological subtype | P value for the subgroups difference | 0.62 | 0.09 | - | 0.67 |
|  | Heterogeneity Cochran’s Q (p value, *I*^2^) | p = 0.33, *I*^2^ = 13% | p = 0.35, *I*^2^ = 9% | - | p = 0.59, *I*^2^ =0% |
